# Long-term prognostic outcomes in patients with haemoptysis

**DOI:** 10.1186/s12931-021-01809-6

**Published:** 2021-08-04

**Authors:** Michele Mondoni, Paolo Carlucci, Giuseppe Cipolla, Matteo Pagani, Francesco Tursi, Alessandro Fois, Pietro Pirina, Sara Canu, Stefano Gasparini, Martina Bonifazi, Silvia Marani, Andrea Comel, Laura Saderi, Sabrina De Pascalis, Fausta Alfano, Stefano Centanni, Giovanni Sotgiu

**Affiliations:** 1 Respiratory Unit, ASST Santi Paolo e Carlo, San Paolo Hospital, Department of Health Sciences, Università degli Studi di Milano, Via A. Di Rudinì n.8, 20142 Milan, Italy; 2 UOC Pneumologia, ASST Lodi, Lodi, Italy; 3 UO Pneumologia ed Endoscopia Toracica, Azienda Ospedaliero Universitaria di Parma, Parma, Italy; 4 UOS Servizio Pneumologia, Ospedale di Codogno, ASST Lodi, Codogno (Lodi), Italy; 5 Lung Disease Unit, Dept of Clinical and Experimental Medicine, University of Sassari, Sassari, Italy; 6 Pulmonary Disease Unit, Department of Internal Medicine, Azienda Ospedali Riuniti, Department of Biomedical Sciences and Public Health, Università Politecnica Delle Marche, Ancona, Italy; 7 UO Medicina Interna, AUSL Modena, Ospedale di Carpi, Carpi (Modena), Italy; 8 UO Pneumologia, Ospedale P. Pederzoli, Peschiera del Garda (Verona), Italy; 9 Clinical Epidemiology and Medical Statistics Unit, Dept of Medical, Surgical and Experimental Sciences, University of Sassari, Sassari, Italy

**Keywords:** Haemoptysis, Recurrence, Lung cancer, Bronchoscopy, Anticoagulant, Antiplatelet, Mortality, Bronchiectasis

## Abstract

**Background:**

Haemoptysis is a challenging symptom that can be associated with potentially life-threatening medical conditions. Follow-up is key in these patients to promptly detect new or misdiagnosed pathologic findings. Few prospective studies have evaluated long-term prognostic outcomes in patients with haemoptysis. Furthermore, the role played by antiplatelet and anticoagulant drugs on mortality and recurrence rates is unclear. The aim of this study was to assess mortality after 18 months of follow-up. Furthermore, the incidence of recurrence and the risk factors for recurrence and death were evaluated (including the role played by anticoagulant and antiplatelet drugs).

**Methods:**

Observational, prospective, multicentre, Italian study.

**Results:**

451/606 (74.4%) recruited patients with haemoptysis completed the 18 months follow-up. 22/604 (3.6%) diagnoses changed from baseline to the end of the follow-up. 83/604 (13.7%) patients died. In 52/83 (62.7%) patients, death was the outcome of the disease which caused haemoptysis at baseline. Only the diagnosis of lung neoplasm was associated with death (OR (95%CI): 38.2 (4.2–347.5); p-value: 0.0001). 166 recurrences were recorded in 103/604 (17%) patients. The diagnosis of bronchiectasis was significantly associated with the occurrence of a recurrence (OR (95% CI): 2.6 (1.5–4.3)); p-value < 0.0001). Anticoagulant, antiaggregant, and anticoagulant plus antiaggregant drugs were not associated with an increased risk of death and recurrence.

**Conclusions:**

Our study showed a low mortality rate in patients with haemoptysis followed-up for 18 months. Pulmonary malignancy was the main aetiology and the main predictor of death, whereas bronchiectasis was the most frequent diagnosis associated with recurrence. Antiplatelet and/or anticoagulant therapy did not change the risk of death or recurrence. Follow-up is recommended in patients initially diagnosed with lower airways infections and idiopathic bleeding.

*Trial registration*: NCT02045394

## Background

Haemoptysis is a challenging symptom that can be associated with potentially life-threatening medical conditions [1, 2]. Recent studies have shown that lung cancer, bronchiectasis, and lower respiratory tract infections are the most frequent aetiologies [2–8]. However, despite an accurate initial work-up, a subgroup of patients with haemoptysis does not have an aetiological diagnosis (*i.e.*, idiopathic or cryptogenic haemoptysis) [2–9]; furthermore, diagnostic changes from the baseline assessment to recurrences have been recently described [2, 9]. In particular, lung cancers and bronchiectasis were subsequently diagnosed in patients with an initial diagnosis of idiopathic haemoptysis and lower respiratory tract infection [2, 10]. In this context, follow-up is key to detecting new or misdiagnosed pathologic findings (*e.g.*, lung malignancy) [2, 6, 8].

Several factors might influence the long-term prognostic outcomes of patients with haemoptysis. Few prospective studies have evaluated the survival rate, the mortality-related risk factors, or the incidence of recurrence and its associated variables [7, 8]. Furthermore, the role played by antiplatelet and anticoagulant drugs is still unclear.

The primary aim of this study was to assess the mortality after 18 months of follow-up; furthermore, the incidence of recurrence and the risk factors for recurrence and death were evaluated. The role of anticoagulant and antiplatelet drugs on these outcomes was also studied.

## Materials and methods

### Study design

This is a secondary analysis of an observational, prospective, multicentre, Italian study aimed at evaluating the epidemiology of haemoptysis in Italy and the diagnostic yield of the most frequently prescribed diagnostic techniques [5]. It was approved by the ethical committees of five Italian participating hospitals and registered at ClinicalTrials.gov (identifier: NCT02045394). Written informed consent was provided by all recruited patients [5], who were followed-up for 18 months.

One month after the recruitment and the first initial assessment, a hospital clinical re-evaluation was scheduled. After three, six, nine, twelve, and eighteen months, a phone call was planned for every patient. At each follow-up visit, information on the occurrence, timing, and severity of recurrences was collected.

In case of a recurrence, a new clinical assessment was performed; data on clinical, radiological, and endoscopic examinations, as well as on symptom management were recorded.

### Patients and interventions

From July 2013 to September 2015, adult (*i.e.*, ≥ 18 years old) patients with haemoptysis requiring an aetiological diagnosis were considered eligible for recruitment [5] and consecutively enrolled. Exclusion criteria were the following: 1) aetiology of haemoptysis already known; 2) refusal to sign the informed consent. The follow-up period lasted from December 2015 to February 2018.

The severity of haemoptysis was graded by the first attending physician. Patients were divided into three groups based on the total amount of blood expectorated in 24 h (h) [5, 7]: mild (*i.e.*, drops of blood to 20 millilitres (ml)/24 h), moderate (*i.e.*, 20–500 ml/24 h), severe (*i.e.*, > 500 ml/24 h).

### Outcome measures

The primary outcome was the survival rate of patients with haemoptysis. Furthermore, the incidence of recurrence was calculated, and the main factors associated with recurrence and mortality were determined. The effectiveness of antiplatelet and anticoagulant drugs on these outcomes was specifically investigated. Changes in the diagnosis of haemoptysis from baseline to the end of follow-up were recorded.

### Statistical analysis

Qualitative and quantitative variables were collected with an ad hoc electronic form. Qualitative variables were described with absolute and relative (percentage) frequencies, whereas quantitative variables were summarised as medians (interquartile ranges, IQR) for their non-parametric distribution. Univariate and multivariate logistic regression analysis was performed to assess the relationships between clinical, demographic, and epidemiological variables and the outcomes death and recurrence. A two-tailed p-value less than 0.05 was considered statistically significant. All the statistical computations were performed with the statistical software STATA version 16 (StatsCorp, Texas, USA).

## Results

An 18 month follow-up assessment was completed by 451 out of 606 (74.4%) patients who were recruited [5].

The initial aetiological diagnoses were previously described [5]. In addition to specific aetiological therapy, bronchial artery embolisation was performed in 13/606 (2.1%) patients, and bronchoscopy was performed with a therapeutic aim (e.g., administration of topical vasoconstriction, Fogarty balloon, endobronchial argon plasma coagulation, and laser therapy) in 99/604 (16.4%) patients. Oral and intravenous tranexamic acid were prescribed in 119/606 (19.6%) patients.

70/606 (11.5%) patients were lost to follow-up, whereas 83/606 (13.7%) died during the study period. Within one month after the enrolment 15/83 (18.1%) patients died. 21/83 (25.3%) patients died between the first and the third month of follow-up, 14/83 (16.9%) between the third and the sixth month, 26/83 (31.3%) between the sixth and the twelfth month, and 7/83 (8.4%) between the twelfth and the eighteenth month.

Death was the outcome of the disease which caused haemoptysis at baseline in 52/83 (62.7%) patients: 42 died due to lung cancer (eight during a recurrence), seven patients for metastatic pulmonary malignancy, two patients for pneumonia, and one for a bronchiectasis exacerbation. One patient, initially diagnosed with idiopathic haemoptysis, died during a recurrence without an identifiable cause of bleeding.

In the univariate analysis, age > 70 years (odds ratio, OR: 9.5; p-value: 0.03), being male r (OR: 2.3; p-value: 0.005), moderate haemoptysis (OR: 1.9; p-value: 0.01), smoking history (≥ 10 pack/years: OR: 3.5; p-value: < 0.0001; ≥ 30 pack/years: OR 1.9; p-values: 0.02), and pulmonary malignancy (OR: 15.6; p-value: < 0.0001) were associated with an increased risk of mortality. In the multivariate analysis, only the diagnosis of lung neoplasm resulted significantly associated with the above-mentioned outcome (OR: 38.2; p-value: 0.0001) (Table 1).

**Table 1:** Demographic and clinical variables predictive of mortality in patients with haemoptysis

	Univariate analysis		Multivariate analysis	
	OR (95% CI)	p-value	OR (95% CI)	p-value
Age (classes)				
< 40 years	Ref	Ref		
40–54 years	0.8 (0.1–9.0)	0.85		
55–70 years	3.3 (0.4–26.4)	0.25		
> 70 years	9.5 (1.3–70.5)	0.03	5.0 (0.8–32.7)	0.10
Sex male	2.3 (1.3–4.1)	0.005	1.3 (0.2–7.4)	0.76
Haemoptysis severity				
Mild	Ref	Ref		
Moderate	1.9 (1.2–3.0)	0.01		
Severe	0.6 (0.1–4.6)	0.61		
Smoking history				
≥ 10 pack/years	3.5 (1.8–6.7)	< 0.0001	0.5 (0.1–3.5)	0.46
≥ 30 pack/years	1.9 (1.1–3.4)	0.02		
Number of recurrences	0.2 (0.1–0.7)	0.009	0.6 (0.2–2.3)	0.46
Severity of recurrences				
Mild	Ref	Ref		
Moderate	3.6 (1.2–10.8)	0.02		
Severe	10.8 (1.6–73.9)	0.02		
Antiplatelet therapy	1.3 (0.8–2.1)	0.37		
Anticoagulant therapy	1.5 (0.8–2.9)	0.25		
Antiplatelet + anticoagulant therapy	1.3 (0.8–2.1)	0.24		
Pneumonia/lung abscess	0.7 (0.3–1.3)	0.20		
Malignancy (primary and metastatic)	15.6 (9.2–26.5)	< 0.0001	38.2 (4.2–347.5)	0.0001
Bronchiectasis	0.1 (0.0–0.5)	0.004	0.3 (0.0–3.7)	0.37
Acute bronchitis	–	–		
Cryptogenic haemoptysis	0.3 (0.1–1.1)	0.08		

From baseline to the end of the follow-up, 22/606 (3.6%) diagnoses changed (Table 2). In particular, pulmonary malignancy was described in four cases initially diagnosed with idiopathic bleeding, in two patients with an exacerbation of chronic obstructive pulmonary disease (COPD) (both with a smoking history > 20 pack/years), and in four patients with pneumonia/lung abscess (two of them active smokers). In 7/22 (32%) patients, the new diagnosis was determined during a recurrence: lung cancer was described in two patients with an initial diagnosis of pneumonia/lung abscess and in one with idiopathic bleeding. In two patients, an upper airways lesion was found after an initial diagnosis of acute bronchitis and idiopathic bleeding. An upper digestive haemorrhage and a pulmonary embolism were diagnosed in two patients initially diagnosed with cryptogenic haemoptysis and pneumonia, respectively.

**Table 2:** Variation in the diagnosis from the initial assessment after 18 months of follow-up

Initial diagnosis	Diagnosis at the end of follow-up	Patient number
Pneumonia/lung abscess	Lung cancer	4
Pneumonia/lung abscess	Pulmonary embolism (with infarction)	2
Pneumonia/lung abscess	Bronchiectasis	1
Cryptogenic haemoptysis	Lung cancer	4
Cryptogenic haemoptysis	Upper airways bleeding disease	1
Cryptogenic haemoptysis	ILD	1
Cryptogenic haemoptysis	Hematemesis	1
COPD exacerbation	Bronchiectasis	2
COPD exacerbation	Pulmonary malignancy	1
COPD exacerbation	Lung cancer	1
Acute bronchitis	Bronchiectasis	1
Acute bronchitis	COPD exacerbation	1
Acute bronchitis	Upper airways bleeding disease	1
Acute bronchitis	ILD	1

*COPD* chronic obstructive pulmonary disease, *ILD* interstitial lung disease

Recurrent episodes of haemoptysis were recorded in 103/606 (17%) patients (Table 3). The median (IQR) number of events was 1 (1–2). Among 103 patients with bronchiectasis, 28 (27.2%) experienced recurrences (45 bleeding events). Recurrences were also recorded in 24/103 (23.3%) with pulmonary malignancy (22/103 (21.4%) with lung cancer (26 events) and 2/103 (1.9%) with pulmonary metastasis (4 events), in 13/103 (12.6%) with pneumonia/lung abscess (21 events). Bronchiectasis and pneumonia/lung abscess related recurrences were mostly mild (20 (71.4%) and 13 (100%) patients, respectively), whereas in case of lung cancer mild to moderate bleeding was found in 10 (45.5%) patients. Two (7.1%) severe recurrences were recorded in patients with both bronchiectasis and lung cancer.

**Table 3:** Haemoptysis recurrences and their severity in 103 patients related to the final diagnosis

Final diagnosis	Events	Patients	Mild	Moderate	Severe
Bronchiectasis, n (%)	45	28 (27.2)	20 (71.4)	6 (21.4)	2 (7.1)
Pulmonary malignancy, n (%)	30	24 (23.3)	11(36.6)	11 (36.6)	2 (0.8)
Lung cancer, n (%)	26	22 (21.4)	10 (45.5)	10 (45.5)	2 (9.1)
Pulmonary metastasis, n (%)	4	2 (1.9)	1 (50.0)	1 (50.0)	0 (0.0)
Pneumonia/lung abscess, n (%)	21	13 (12.6)	13 (100)	0 (0.0)	0 (0.0)
COPD (exacerbation), n (%)	13	7 (6.8)	7 (100.0)	0 (0.0)	0 (0.0)
Acute bronchitis, n (%)	11	7 (6.8)	7 (100.0)	0 (0.0)	0 (0.0)
Cryptogenic haemoptysis, n (%)	10	6 (5.8)	5 (83.3)	1 (16.7)	0 (0.0)
Upper airways bleeding disease, n (%)	8	4 (3.9)	2 (50.0)	2 (50.0)	0 (0.0)
Post-tuberculosis sequelae, n (%)	8	3 (2.9)	2 (66.7)	1 (33.3)	0 (0.0)
Other pulmonary/bronchial vascular lesion, n (%)	5	2 (1.9)	1 (50.0)	0 (0.0)	1 (50.0)
Pulmonary embolism, n (%)	5	2 (1.9)	1 (50.0)	1 (50.0)	0 (0.0)
Atypical mycobacteriosis, n (%)	2	1 (1.0)	1 (100.0)	0 (0.0)	0 (0.0)
Active tuberculosis, n (%)	2	2 (1.9)	1 (50.0)	1 (50.0)	0 (0.0)
Iatrogenic or traumatic, n (%)	2	1 (1.0)	1 (100.0)	0 (0.0)	0 (0.0)
Tracheal granuloma, n (%)	2	1 (1.0)	1 (100.0)	0 (0.0)	0 (0.0)
Hematemesis, n (%)	1	1 (1.0)	0 (0.0)	1 (100.0)	0 (0.0)
Interstitial lung disease, n (%)	1	1 (1.0)	1 (100.0)	0 (0.0)	0 (0.0)

*COPD* chronic obstructive pulmonary disease

The univariate analysis showed that bronchiectasis was significantly associated with the occurrence of a recurrence (OR: 2.6); p-value < 0.0001) (Table 4). Anticoagulant, antiaggregant, and anticoagulant plus antiaggregant drugs were not significantly associated with an increased risk of mortality or recurrence (Table 5).

**Table 4:** Demographic and clinical variables predictive of recurrence in patients with haemoptysis

	Univariate analysis		Multivariate analysis	
	OR (95% CI)	p-value	OR (95% CI)	p-value
Age (years)				
< 40	Ref.	Ref.		
40–54	1.3 (0.4–4.4)	0.63		
55–70	1.6 (0.5–5.0)	0.41		
> 70	1.9 (0.7–5.7)	0.23		
Sex male	0.9 (0.6–1.3)	0.51		
Severity of haemoptysis				
Mild	Ref.	Ref.		
Moderate	1.2 (0.8–2.0)	0.39		
Severe	1.2 (0.8–2.0)	0.35		
Smoking history				
≥ 10 pack/years	0.7 (0.4–1.1)	0.09		
≥ 30 pack/years	0.9 (0.5–1.6)	0.74		
Antiplatelet therapy	0.9 (0.5–1.5)	0.69		
Anticoagulant therapy	1.1 (0.6–2.2)	0.75		
Antiplatelet + anticoagulant therapy	1.0 (0.6–1.6)	0.98		
Pneumonia/lung abscess	0.7 (0.4–1.3)	0.25		
Malignancy (primary and metastatic)	1.1 (0.6–1.9)	0.79		
Bronchiectasis	2.6 (1.5–4.3)	< 0.0001		
Acute bronchitis	0.6 (0.3–1.3)	0.18		
Cryptogenic haemoptysis	1.0 (0.5–2.1)	0.94

**Table 5:** Role played by antiplatelet and anticoagulant therapy, alone and combined, on recurrence and mortality in patients with haemoptysis

Antiplatelet and anticoagulant therapy	No	Yes	p-value
Recurrence, n (%)	68 (20.1)	35 (18.8)	0.72
Recurrence severity, n (%)			
Mild	49 (72.1)	25 (71.4)	0.46
Moderate	17 (25.0)	7 (20.0)	
Severe	2 (2.9)	3 (8.6)	
Median (IQR) n. recurrence events	1 (1–2)	1 (1–2)	0.12
Deaths after 18 months of follow-up, n (%)	50 (12.6)	33 (16.0)	0.24
Deaths for the disease which caused haemoptysis, n (%)	31 (62.0)	21 (63.6)	1.00
**Antiplatelet therapy**			
Recurrence, n (%)	80 (20.2)	23 (18.0)	0.58
Recurrence severity, n (%)			
Mild	59 (73.8)	15 (65.2)	0.16
Moderate	19 (23.8)	5 (21.7)	
Severe	2 (2.5)	3 (13.0)	
Median (IQR) n. recurrence events	1.5 (1–2)	1 (1–2)	0.04
Deaths after 18 months of follow-up, n (%)	60 (13.0)	23 (16.0)	0.37
Deaths for the disease which caused haemoptysis, n (%)	35 (58.3)	17 (73.9)	0.19
**Anticoagulant therapy**			
Recurrence, n (%)	91 (19.7)	12 (19.7)	0.98
Recurrence severity, n (%)			
Mild	64 (70.3)	10 (83.3)	0.85
Moderate	22 (24.2)	2 (16.7)	
Severe	5 (5.5)	0 (0.0)	
Median (IQR) n. recurrence events	1 (1–2)	2 (1–2)	0.66
Deaths after 18 months of follow-up, n (%)	71 (13.2)	12 (18.5)	0.24
Deaths for the disease which caused haemoptysis, n (%)	47 (66.2)	5 (41.7)	0.10

## Discussion

To the best of our knowledge, this is the largest prospective study describing the long-term prognostic outcomes (18 months) of patients with haemoptysis.

An overall mortality rate of 13.7% was found; the number of deaths increased from 18.1 to 31% after one year of follow-up and then decreased to 8.4% at the end of the study period. Most of the deaths occurring during follow-up were related to the aetiology that caused the haemoptysis, with pulmonary neoplasms being the leading cause.

Malignancy, which was reported as the most frequent aetiology in patients with haemoptysis in several studies [3–6, 8, 11], represents the only significant predictor of mortality in our study. Haemoptysis related to bronchiectasis, lower respiratory tract infections, and other less frequent aetiologies was not associated with an increase in mortality.

Two previous prospective studies with a small sample size and a follow-up period of 1.8–2.7 years described a slightly higher mortality rate (19.5–22%) [7, 8], mainly driven by malignancies [8]. More heterogeneous findings were reported by recently published European retrospective studies where lung cancer was the major cause of death, but the mortality rate ranged from 5.9 to 27% [2, 6, 10]. Petersen et al. showed that older age, previous diagnosis of lung cancer, current/previous smoking history, and concomitant lung diseases are independent risk factors of death [10]. Notably, cryptogenic haemoptysis accounted for 80.5% of the diagnoses in this cohort, suggesting a suboptimal or difficult-to-perform diagnostic assessment [10].

The recurrence rate was 17%, with bronchiectasis being the leading cause (27.2%), and the most important predictor of recurrence. Bronchiectasis recurrences were mainly mild in contrast with those caused by lung malignancies (*i.e.*, the second most frequent cause of recurrence), which were equally mild and moderate.

Few recent retrospective data on this topic are available in the scientific literature. In keeping with our findings, Fidan et al. and Ryuge et al. found bronchiectasis as the most frequent diagnosis in recurrent hemoptysis [12, 13]. Abdulmalak et al. described recurrence rates of 16.6% and 16.1% during a three-year follow-up period in 2008 and 2009, respectively [2]. Cryptogenic haemoptysis, lower respiratory tract infection, and lung cancer were the most common aetiologies of recurrences, with cryptogenic haemoptysis representing 50% of all causes.

Choi et al., who described a recurrence rate of 19.1%, demonstrated that aspergillosis, active bleeding, and blood clots during bronchoscopy during the first evaluation were significantly associated with the risk of recurrence; however, they analysed only patients with mild hemoptysis [14]. Similar findings were described by Lee et al., who found that active bleeding during bronchoscopy, smoking history > 40 years, and hypertension were the main predictors of recurrence. Notably, the aetiology was not evaluated in this study [15].

As suggested by Lee et al., recurrences should alert physicians to undetected pathological findings [16, 17].

In seven patients of our cohort, a recurrence was associated with a new clinical and radiological assessment and to an aetiological change. Three patients initially diagnosed with idiopathic bleeding or pneumonia were subsequently diagnosed with lung cancer, in contrast to the study by Tsoumakidou et al., where lung cancer was not diagnosed in any patients with an initial aetiology other than lung cancer [7]. On the contrary, Abdulmalak et al. described the highest rate of lung cancer detection during the follow-up of patients with a diagnosis of respiratory infection (10.4%) at the baseline evaluation [2].

In our study, four patients initially diagnosed with acute bronchitis and COPD exacerbation based on clinical and chest X-ray findings were subsequently diagnosed with bronchiectasis.

The present scientific evidence suggests the importance of a clinical and radiological follow-up in patients with bleeding of unknown origin and related to an acute lower respiratory tract infection, as well as a more accurate radiological assessment (*i.e.*, chest CT) in patients with haemoptysis and risk factors for bronchiectasis [18].

Very little data could be retrieved on the impact of antiplatelet or anticoagulant therapies on long-term prognostic outcomes. In a large retrospective cohort study, Lee et al. failed to demonstrate a potential role of aspirin on recurrence [15]. Similar findings were shown by Ryuge et al., who studied the mechanism of haemoptysis relapse in patients who underwent BAE; they demonstrated that antiplatelet and anticoagulant therapy did not increase recanalisation, i.e. the most frequent mechanism underlying re-hemoptysis [13]. We have prospectively demonstrated that these drugs, individually or in combination, did not change the risk of death and recurrence.

Some study limitations can be found. The observational nature cannot help discriminate the role played by some medical conditions for the background noise of confounders. However, for ethical reasons, it is currently the best methodological approach. Differences in some standard operating procedures in the recruited centres could affect some results in terms of diagnostic accuracy. However, the recruited hospitals were national reference centres, and thus, operational variability should be not relevant. Subgroup analyses by aetiology could have affected the statistical power of some findings; future studies focused on specific aetiologies could confirm our novel results.

## Conclusions

In conclusion, our study shows a low mortality rate in patients with haemoptysis followed-up for a long period. Pulmonary malignancy is the main aetiology and the main predictor of death in these patients, whereas bronchiectasis is the most frequent diagnosis associated with recurrence. Antiplatelet and/or anticoagulant therapy do not change the risk of death or recurrence. Follow-up is recommended in patients initially diagnosed with lower airways infections and those with idiopathic bleeding, in order to detect new or misdiagnosed lung malignancies.

## Data Availability

The datasets used and/or analysed during the current study are available from the corresponding author on reasonable request.

## Abbreviations

ml: Millilitres
IQR: Interquartile range
COPD: Chronic obstructive pulmonary disease
